# Reply to Elgoyhen et al. Comment on “De Rosa et al. Hearing Loss: Genetic Testing, Current Advances and the Situation in Latin America. *Genes* 2024, *15*, 178”

**DOI:** 10.3390/genes15111403

**Published:** 2024-10-30

**Authors:** Katherina Walz

**Affiliations:** 1 Instituto de Química Biológica de la Facultad de Ciencias Exactas y Naturales (IQUIBICEN) CONICET, Facultad de Ciencias Exactas y Naturales, Universidad de Buenos Aires, Buenos Aires C1428EHA, Argentina; kwalz@med.miami.edu; 2 John P. Hussman Institute for Human Genomics, Miller School of Medicine, University of Miami, Miami, FL 33136, USA; 3 John T. Macdonald Foundation Department of Human Genetics, Miller School of Medicine, University of Miami, 1501 NW 10th Avenue, BRB-418 (M-860), Miami, FL 33136, USA

First, thanks to Elgoyhen et al. for presenting an elaborate discussion [1] on our published review “Hearing Loss: Genetic Testing, Current Advances and the Situation in Latin America”. In their comment, Elgoyhen et al. concur with our conclusion that the implementation of sequencing technologies in Latin American (LATAM) countries is far from optimal. Despite commendable efforts by institutions, such as those listed in Table 2 of their comment (from three LATAM countries), the current biomedical infrastructure in LATAM remains a resource-constrained environment, challenging the widespread integration of genomics and bioinformatics into public health systems.

Elgoyhen et al. argue that although our review contains “a comprehensive wealth of data”, it would benefit from additional information. Thus, they have provided references related to genetics studies focusing on stereocilin (*STRC*), which we omitted due to our focus on profound hearing loss. Nevertheless, they are a welcome addition since they complement the pool of genetic variations related to hearing loss in the Hispanic population. Furthermore, their comment mentions five additional references (three of their additional references were cited in our review paper) supporting the increasing adoption of NGS in molecular diagnosis of hearing loss in LATAM. Apart from the three citations describing syndromic hearing loss cohorts [2,3,4], which were therefore not discussed, the additional references [5,6] are very valuable. Anyway, we feel these numbers, despite the expected omissions of further reports not included in our review, reinforce the main point of our paper, well put by Elgoyhen et al.: “there is still a long road ahead to bring genetic counseling and genetic/genomic studies to Latin Americans”.

In conclusion, we did not question the high quality of research and clinical genetics being conducted in Latin America, nor did we deny the existence of centers of excellence focusing on hearing loss (some of them listed in Elgoyhen et al.). However, we reinforced the need to increase resources and accessibility to these new technologies for the benefit of the population in the LATAM region.
